# The Application of Neuroimaging to Healthy and Diseased Brains: Present and Future

**DOI:** 10.3389/fneur.2017.00061

**Published:** 2017-02-24

**Authors:** Jan Kassubek

**Affiliations:** ^1^Department of Neurology, University of Ulm, Ulm, Germany

**Keywords:** neuroimaging, MRI, brain imaging, translational neuroimaging, challenges

Brain imaging has become a core technical element of the clinical work-up in patients with neurological and psychiatric diseases including nervous system manifestations of systemic diseases. Beyond (but closely connected to) that clinical approach, the opportunities of brain imaging have radically changed our assessment of brain structure and function in health and also their alterations in association with disease. Neuroimaging helps to understand how the brain and the other parts of the nervous system work and what structural or functional alterations may be associated with a given clinical presentation of a disease or medical condition. This aspect of clinicoradiological correlations—which is intrinsically tied to neuroanatomy—needs to be addressed both in a clinical and in a neuroscientific context in order to deepen our understanding of what can be visualized by imaging of the nervous system. To reach this goal, the approach has to be integrative and multidisciplinary in nature, collecting clinically oriented researchers and neuroscientists of different areas. The application to the healthy and diseased brain’s texture is independent of the neuroimaging modality—although magnetic resonance imaging seems to be the most promising technical tool to decode brain structure and function, given its development over the recent decade, other brain mapping techniques including but not limited to electric/magnetic source imaging or radioligand imaging have important further aspects to add to the assessment of the working brain. In addition, approaches with correlation analyses with multimodal data from other technical tools or clinical parameters (including genomics, metabolomics, and many more) for the definition of the investigated phenotype will remain one important element for the further establishment of neuroimaging metrics. Finally, translational neuroimaging research will help to further expand our systematic knowledge of the physiology and pathology in the human and animal neural system. In this integrative approach, neuroimaging will be the core element to elaborate the concept of computer-based neuroanatomy and pathoneuroanatomy. Such a framework is also essential for studies on functional reserve and compensation in aging and disease and the development of the growing brain. Advanced neuroimaging is *per se* “descriptive” as a technique, but has recently moved away from mere qualitative characteristics of the brain’s structural and functional organization to quantitative measures and predictive models that are becoming practical for use as biological markers or for treatment monitoring in disease (1). As such, neuroimaging techniques will not only guide clinical diagnosis but will be a part of the concepts of personalized medicine also with respect to a patient’s prognosis.

There is a striking analogy between the organization of the clinical and the neuroscientific researchers themselves on the one hand and the understanding of the brain’s system on the other hand. Within the neuroscientific community, a trend for bottom-up initiatives is emerging, starting with small-scale projects by single groups that expand upon existing collaborations of researchers and infrastructures and develop to grand-scale projects for which the European *Human Brain Project* (2) is only one example out of many international initiatives. Joined forces in “meso-scale” collaborations with a focus on single brain functions might be a solution to the existing challenges by centralizing around self-organized groups of researchers with specialized expertise (3). That way, a “naturally” growing number of worldwide neuroimaging collaborations build the era of “big data science” for neuroimaging.

With respect to the understanding of the brain, a conceptual shift has occurred in the last decade toward using network-based, rather than region-based, approaches to characterize brain structure and function and its alterations in disease (4). For aging, a generative framework for computationally modeling the connectome over the human life span has been proposed in the understanding that the human connectome gradually shifts from an “anatomically driven” organization to one that is more “topological” (5). Advances in connectomics have led to a synthesis of perspectives regarding the brain’s functional organization that reconciles classical concepts of localized specialization with an appreciation for properties that emerge from interactions across distributed functional networks, as a more comprehensive framework for understanding neural mechanisms of normal function and disease, with contributions to modern concepts for the treatment (1).

In this framework, single-center studies and region-based investigations, together with multisite studies and network-based investigations, have their roles in the ongoing efforts to decipher the nervous system’s structural and functional interplay by neuroimaging, each using various methodological imaging modalities. Neuroimaging has to be seen in a close context with neuroanatomy, including a growing variety of specified brain atlases and the recent developments in mapping the microscopical organization of the human cerebral cortex with observer-independent parcellations and the concept of probabilistic mapping, as prerequisites to understand the organizational principles of the brain at its different spatial scales with cellular and even subcellular components (6). The use of the technical opportunities in humans should be complemented by the explicit consideration of the embodiedness of the brain and the embeddedness of humans, as Kotchoubey et al. (7) stated, in order to aim at “the development of an explicit methodology of integrative human neuroscience, which will not only link different fields and levels but also help in understanding clinical phenomena.”

The new section “Applied Neuroimaging” of *Frontiers in Neurology* will aim at the integration of research both on clinical and on neuroscientific grounds with all modalities of neuroimaging in order to be a forum in the promising field of neuroimaging applications to advanced structural and functional mapping of the nervous system. These technical tools will here be recognized as new approaches to the nervous system’s organization during the life span, its pathology in disease, as biological markers, and as a guide in the individualization of patient management and in the design of new interventions to improve clinical outcomes.

## Author Contributions

The author confirms being the sole contributor of this work and approved it for publication.

## Conflict of Interest Statement

The author declares that the research was conducted in the absence of any commercial or financial relationships that could be construed as a potential conflict of interest.
